# Potential impact of alcohol calorie labelling on the attitudes and drinking behaviour of hazardous and low-risk drinkers in England: a national survey

**DOI:** 10.1136/bmjopen-2024-087491

**Published:** 2024-08-31

**Authors:** Andrew Steptoe, Florence Sheen, Rana Conway, Clare Llewellyn, Jamie Brown

**Affiliations:** 1 Department of Behavioural Science and Health, University College London, London, UK; 2 School of Sport, Exercise & Health Sciences, Loughborough University, Loughborough, UK; 3 Behavioural Science and Health, University College London, London, UK

**Keywords:** behavior, ethanol, health surveys, obesity

## Abstract

**Abstract:**

**Objectives:**

We investigated the hypothetical impact of mandatory alcohol calorie labelling, comparing non-drinkers, low-risk and hazardous drinkers in terms of attitudes, knowledge about calorie content and hypothetical behaviour changes should labelling be introduced.

**Design:**

Cross-sectional national telephone survey.

**Setting:**

Community-dwelling adults in England between November 2022 and January 2023.

**Participants:**

Data were collected from 4683 adults >18 years, of whom 24.7% were non-drinkers; 77.6% of alcohol drinkers were categorised as low-risk and 22.4% as hazardous drinkers according to the Alcohol Use Disorders Identification Test questionnaire.

**Primary outcome measures:**

Attitudes to alcohol calorie labelling in shops and supermarkets and in hospitality venues, knowledge of the calorie content of alcoholic beverages (beer, wine, cider and spirits) and changes in drinking practices if calorie labelling was introduced.

**Results:**

Comparisons were made between non-drinkers, low-risk drinkers and hazardous drinkers, with analyses adjusted for age, gender, ethnicity, socioeconomic status and education. Attitudes to calorie labelling were generally positive, but were less favourable among alcohol drinkers than non-drinkers. Hazardous drinkers were more accurate in their estimations of the calorie content of wine, cider and spirits than non-drinkers (p<0.0001). Overall, 46.4% of drinkers indicated that they would change their drinking patterns if calorie labelling was introduced, and this response was more common among hazardous than low-risk drinkers (OR=1.43, 95% CI 1.199 to 1.699), adjusting for age, gender, ethnicity, socioeconomic status and education. Compared with low-risk drinkers, hazardous drinkers stated that they would be more likely to drink fewer alcoholic beverages, to drink alcohol less often, to choose lower calorie drinks and to do more exercise (adjusted OR 1.27, 1.009 to 1.606).

**Conclusions:**

A sizeable proportion of hazardous drinkers indicated that they would change their consumption practices if mandatory calorie labelling was introduced. Promoting more positive attitudes to calorie labelling might lead to stronger intentions to reduce consumption. Mandatory calorie labelling of alcoholic beverages may make a modest contribution to energy intake and the maintenance of health weight, particularly among heavier drinkers.

**STRENGTHS AND LIMITATIONS OF THIS STUDY**

- We collected data from a national representative population sample of adults with levels of alcohol consumption comparable to those in the Health Survey for England.
- Alcohol consumption levels were assessed using a validated questionnaire.
- The study was cross-sectional and findings are based on self-report.
- The study sample included relatively few individuals reporting very high levels of alcohol consumption.
- We had no information about body weight and adiposity, and these are relevant to the issue of total energy intake.

## Introduction

 Excessive alcohol consumption is a substantial public health issue, and it is estimated that 28% of men and 15% of women in England regularly consume more than is recommended in current UK guidelines for low-risk drinking.^1 2^ Alcoholic drinks above 1.2% alcohol by volume (ABV) were exempt from legislation of mandatory calorie labelling for non-prepackaged food and soft drink products sold by large food businesses implemented in April 2022.^3^ However, around 9% of calories consumed by men and 5% by women can be attributed to alcoholic beverages.^4 5^ Observational studies suggest a dose-response association between alcohol intake and greater adiposity.^6^ Introducing mandatory calorie labelling on alcohol drinks could positively affect the prevalence of these issues as well as influencing high-risk alcohol consumption.^7^

There is limited evidence from real-world studies of alcohol calorie labelling on consumer behaviour.^8^ Short-term experiments on alcohol calorie labelling have shown mixed findings, with some evidence for reduced purchasing.^9 10^ Other studies have assessed changes in motivation to drink alcohol in different labelling conditions following online randomisation or pseudo-randomisation.^11 12^ Results have been variable, with some evidence for favourable changes in purchasing intentions or motivation to drink less following calorie labelling.^13^,^15^

The likely impact of calorie labelling on alcohol consumption appears to be small.^16^ One reason may be that alcohol consumption patterns have not been taken into account. The potential benefits of energy labelling will be greater among people with high alcohol intake compared with low-risk drinkers, since the contribution of alcohol to total energy intake will be greater. However, few studies have compared heavy and lighter drinkers, or have assessed typical alcohol consumption patterns. The present survey explicitly compared hazardous (increasing and higher risk drinkers) with low-risk drinkers categorised on the well-established Alcohol Use Disorders Identification Test (AUDIT).^17^ We analysed data from a large national survey to investigate attitudes to alcohol calorie labelling and estimates of behaviour change if labelling was introduced. Data were collected by telephone rather than online, minimising the known selection biases in internet-based sampling that reduce representativeness in surveys.^18^ We measured a range of possible behaviour changes that might follow alcohol calorie labelling, since these might not only include modifications in alcohol consumption, but also in food intake and physical activity.^16^ Additionally, we assessed awareness of the calorie content of common alcoholic beverages, because underestimation or overestimation is relevant to the understanding of health risks, and might influence responses to mandatory labelling.^19^

## Methods

### Study design and participants

We commissioned questions on alcohol calorie labelling to be added to the Ipsos Mori Omnibus Survey as part of the Alcohol Toolkit Study, a detailed study that has collected monthly data since 2014 to monitor and understand population-wide influences on alcohol use in adults (18+) living in England (https://www.alcoholinengland.info/graphs/monthly-tracking-kpi). The Omnibus Survey collects data by telephone and uses random sampling at the area level plus quota sampling at the household level to derive the study sample. Data were collected from participants who provided responses to the survey in November 2021, December 2021 or January 2022. Of the 4901 individuals who participated, 218 did not provide data that allowed AUDIT scores to be computed, leaving an analytic sample of 4683.

### Patient and public involvement

Patients and the public were not involved in the design, or conduct, or reporting, or dissemination plans for this research.

### Measures

We collected information about alcohol use with the AUDIT, a self-report 10-item questionnaire originally developed by the WHO to detect harmful alcohol consumption. Scores can range from 0 to 40, with higher ratings indicating higher risk of alcohol dependence. Standard cut-points distinguish low-risk drinkers (1–7) from those with hazardous (8–15), harmful (16–19) and possible alcohol dependence (≥20) drinking levels.^17^ The number of respondents in the higher categories was small (2.1%), so they were combined with those scoring 8–15 to create the hazardous drinking category in the analyses. Individuals with scores >0 and ≤7 were categorised as low-risk drinkers.

The measures of attitudes and changes in behaviour were adapted from those developed by Robinson *et al*,^15^ and are presented in online supplemental file. Attitudes were measured by assessing agreement with three statements: ‘Alcohol calorie labelling would be useful’, ‘Alcohol calorie labelling should be provided in shops and supermarkets’ and ‘Alcohol calorie labelling should be provided in pubs, bars and restaurants’. These were rated on a 5-point Likert scales (from 1=*strongly disagree* to 5=*strongly agree*). The mid-point (3) was labelled ‘neither agree or disagree’ which we grouped with responses 1 and 2, creating categories of agreement (4 and 5) and disagreement (1–3).

Knowledge of the calorie content of alcoholic beverages was assessed by asking participants to estimate the number of calories of four common beverages in standard servings: a pint of beer (586 mL, 4% strength), a medium glass of white wine (175 mL, 13% strength), a pint of cider, (586 mL, 4.5% strength) and a single measure of gin or vodka (25 mL). Answers were given in 50 kcal ranges, with seven response options ranging from 0 to 49 to 300+ kilocalories (kcal). Participants could also answer ‘don’t know’ or ‘refuse to answer’. A 50 kcal range was assigned as the correct response for each beverage, namely 568 mL beer (4% ABV, 150–199 kcal), 568 mL cider (4.5% ABV, 200–249 kcal), 175 mL wine (13% ABV, 100–149 kcal) and 25 mL spirits (40% ABV, 50–99 kcal). This categorisation was based on Food Composition tables^20^ and a search of UK supermarket and popular alcohol brand websites. To take account of estimates near the borders of categories, we coded answers as correct if they were in the precise category and in the immediately adjoining categories.

We evaluated hypothetical changes in behaviour if alcohol calorie labelling was introduced by asking participants the question: ‘If calorie information was provided on alcoholic drinks, which of the following would you do?’ with yes/no answers to the following: ‘I would change my drinking’, ‘I would drink fewer alcoholic drinks’, ‘I would drink less often’, ‘I would choose lower calorie alcoholic drinks’, ‘I would choose smaller serving sizes of alcoholic drinks’, ‘I would eat less (eg, smaller meals or fewer snacks)’, ‘I would do more exercise’, ‘None of these’ and ‘Don’t know’. We hypothesised that hazardous drinkers would be more likely to indicate that they would change their behaviour if calorie labelling was introduced than would low-risk drinkers.

Age, gender and other sociodemographic data were collected by self-report. Ethnicity was recoded into White British and other for the purposes of analysis. Socioeconomic position was based on the Standard Occupational Code 2020 as included in the National Census, with categorisation into A/B, C1, C2 and D/E, where A/B indicates higher and intermediate managerial, administrative, and professional occupations, C1 indicates supervisory, clerical and junior managerial, administrative, professional occupations, C2 includes skilled manual occupations, and D/E semi-skilled and unskilled manual occupations. Educational attainment was based on qualifications and participants were allocated to four groups: none, GCSE/O level (primary education), A level/equivalent (high school qualifications) and degree.

### Statistical analyses

The primary analyses were based on the division of respondents into groups based on AUDIT scores, first comparing non-drinkers with drinkers, and then non-drinkers with low-risk drinkers and hazardous drinkers. There were no missing data on alcohol-related variables. The demographic characteristics of non-drinkers and drinkers, and the differences between hazardous and low-risk drinkers were compared using χ^2^ tests and analysis of variance for categorical and continuous variables, respectively. Logistic regression was used to compare non-drinkers, low-risk and hazardous drinkers on attitudes and knowledge of calorie content. Results are presented as raw percentages, and ORs adjusted for age, gender, ethnicity, occupational class and education with 95% CIs, with non-drinking as the reference category. Hypothetical changes in behaviour in response to alcohol calorie labelling were compared in low-risk (reference category) and hazardous drinkers. In sensitivity analyses, knowledge of calorie content was reanalysed with exact rather than broader categorisation of estimates.

## Results

The study sample consisted of 2264 men and 2419 women (table 1). There were 1155 (24.7%) non-drinkers and 3528 (75.3%) alcohol drinkers, of whom 2736 were classified as low-risk (77.6%) and 792 (22.4%) as hazardous drinkers. Men were more likely to drink alcohol than women, and were more likely than women to be hazardous than low-risk drinkers. Age averaged 50.95±(SD) 19.06 years. Alcohol drinkers and non-drinkers did not differ in age, but hazardous drinkers were an average 8.42 years younger than low-risk drinkers (p<0.0001). The majority of respondents classified themselves as white British (79.4%). Participants belonging to ethnic minority groups were less likely to be drinkers. Alcohol consumption was positively associated with education and socioeconomic position, as was hazardous drinking. The AUDIT scores averaged 3.50±1.81 among low-risk and 11.30±3.95 among people in the hazardous drinking category.

**Table 1:** Participant characteristics

	Complete sample	Non-drinkers	Alcohol drinkers	P value	Low-risk drinkers	Hazardous drinkers	P value
Men	2264 (48.3%)	515 (44.6%)	1749 (49.6%)	<0.004	1240 (45.3%)	509 (64.3%)	<0.0001
Women	2419 (51.7%)	640 (55.4%)	1779 (50.4%)		1496 (54.7%)	283 (35.7%)	
Age (years)	50.95±19.06	50.65±21.09	51.05±18.40	0.54	52.96±18.43	44.54±16.72	<0.0001
Ethnicity							
White British	3716 (79.4%)	765 (66.2%)	2951 (83.6%)	<0.0001	2274 (83.1%)	677 (85.5%)	0.127
Other categories	967 (20.6%)	390 (33.8%)	577 (16.4%)		462 (16.9%)	115 (14.5%)	
Education qualifications							
None	829 (17.7%)	300 (26.0%)	529 (15.0%)	<0.0001	458 (16.7%)	71 (9.0%)	<0.0001
GCSE/O level	921 (19.7%)	231 (20.0%)	690 (19.6%)		531 (19.4%)	159 (20.1%)	
A level/equivalent	1216 (26.0%)	288 (24.9%)	928 (26.3%)		693 (25.3%)	235 (29.7%)	
Degree	1717 (36.7%)	336 (29.1%)	1381 (39.1%)		1054 (38.5%)	327 (41.3%)	
Occupational class							
A/B	1073 (24.7%)	199 (19.2%)	874 (26.5%)		655 (25.7%)	219 (29.3%)	<0.0001
C1	1710 (39.4%)	323 (31.1%)	1387 (42.0%)		1075 (42.1%)	312 (41.8%)	
C2	717 (16.5%)	171 (16.5%)	546 (16.5%)		402 (15.7%)	144 (19.3%)	
D/E	839 (19.3%)	346 (33.3%)	493 (14.9%)	<0.0001	421 (16.5%)	72 (9.6%)	
AUDIT scores	4.05±4.18	0	5.33±4.05	<0.0001	3.50±1.81	11.30±3.95	<0.0001

AUDITAlcohol Use Disorders Identification Test

### Attitudes to alcohol calorie labelling

Attitudes to alcohol calorie labelling were generally positive, with 57.9% indicating that it would be useful, 63.6% agreeing that it should be provided in shops and supermarkets and 51.7% that it should be available in hospitality venues (pubs, bars and restaurants). Women were more likely than men to agree that labelling would be useful, but men and women did not differ in attitudes to whether labelling should be provided at points of sale (online supplemental table 1). Younger respondents were more likely to find calorie labelling useful and to state that it should be provided in shops and supermarkets, while ethnic minority participants were more positive in all attitudes. Ratings of the usefulness of calorie labelling and its provision in shops and supermarkets were positively correlated with socioeconomic status.

There was no difference in the proportion of non-drinkers and alcohol drinkers who agreed that alcohol calorie labelling would be useful (table 2). However, attitudes to the provision of labelling in shops and supermarkets, and in hospitality venues, were markedly lower among alcohol drinkers. After adjustment for covariates, both low-risk and hazardous drinkers were less likely than non-drinkers to agree that alcohol calorie labelling should be provided at points of sale. The extent of agreement did not vary with level of alcohol consumption.

**Table 2:** Attitudes to the introduction of alcohol calorie labelling

Attitude statement	Group	AgreementN (%)	Adjusted OR (95% CI)	P value
Alcohol calorie labelling would be useful	Non-drinkers	585 (57.5%)	1	
	Low-risk	1574 (58.7%)	1.10 (0.936 to 1.289)	0.25
	Hazardous	449 (57.1%)	1.04 (0.844 to 1.278)	0.72
Alcohol calorie labelling should be provided in shops and supermarkets	Non-drinkers	724 (68.9%)	1	
	Low-risk	1674 (62.1%)	0.77 (0.649 to 0.906)	0.002
	Hazardous	480 (61.4%)	0.73 (0.586 to 0.899)	0.003
Alcohol calorie labelling should be provided in pubs, bars and restaurants	Non-drinkers	647 (62.0%)	1	
	Low-risk	1324 (49.3%)	0.65 (0.551 to 0.758)	<0.0001
	Hazardous	362 (46.3%)	0.55 (0.449 to 0.678)	<0.0001

Note: odds ratiosORs adjusted for age, gender, ethnicity, occupational class, and education.

### Knowledge of calorie content of alcoholic beverages

The distribution of estimates of calorie content of beer, white wine, cider and spirits in relation to drinking is detailed in online supplemental table 2. Overall, the greatest accuracy was for spirits (51.5% correct), followed by white wine (41.3%), cider (33.5%) and beer (28.1%). Non-drinkers were more likely to state that they did not know the calorie content than were drinkers. Only a minority of respondents underestimated calorie content, but overestimation was common (eg, 5.9% of hazardous drinkers underestimated the calorie content of beer while 44.8% overestimated).

Among people who provided an estimate of calorie content, there were no differences in accuracy between men and women for any category of beverage (online supplemental table 3). Although none of the demographic factors related to knowledge about beer, there were pronounced socioeconomic gradients for wine and cider, with people in higher categories being more accurate.

Compared with non-drinkers, hazardous drinkers were more likely to be accurate in their estimations of calorie content (table 3). The adjusted ORs ranged from 1.27 (95% CI 1.003 to 1.616) for beer to 1.92 (95% CI 1.469 to 2.495) for spirits. Low-risk drinkers were more accurate than non-drinkers for cider but not for other beverages. The proportion of hazardous drinkers who were correct in their estimates was greater than for low-risk drinkers for white wine (adjusted OR 1.45, 95% CI 1.196 to 1.753) and spirits (adjusted OR 1.77, 95% CI 1.418 to 2.207).

**Table 3:** Knowledge of calorie content of alcoholic beverages

Beverage	Alcohol group	Correct estimateN (%)	Adjusted OR (95% CI)	P value
Beer (pint, 4% ABV)	Non-drinkers	240 (35.5%)	1	
	Low-risk	794 (36.9%)	1.09 (0.893 to 1.322)	0.41
	Hazardous	281 (41.1%)	1.27 (1.003 to 1.616)	0.048
White wine (175 mL, 13% ABV)	Non-drinkers	318 (47.3%)	1	
	Low-risk	1190 (53.9%)	1.17 (0.968 to 1.410)	0.10
	Hazardous	426 (63.3%)	1.69 (1.333 to 2.136)	<0.0001
Cider (pint, 4.5% ABV)	Non-drinkers	249 (37.4%)	1	
	Low-risk	981 (46.5%)	1.33 (1.096 to 1.618)	0.004
	Hazardous	341 (51.3%)	1.51 (1.189 to 1.912)	<0.0001
Spirits (25 mL, 40% ABV)	Non-drinkers	438 (66.2%)	1	
	Low-risk	1441 (68.0%)	1.08 (0.884 to 1.321)	0.45
	Hazardous	532 (79.2%)	1.92 (1.469 to 2.495)	<0.0001

Note: odds ratiosORs adjusted for age, gender, ethnicity, occupational class, and education.

Analysis of respondents who provided an estimate.

ABV, alcohol by volume

### Hypothetical behaviour change with alcohol calorie labelling

Overall, 46.4% of alcohol consumers indicated that they would change their consumption patterns if calorie labelling was introduced. This response was more common among women than men, younger drinkers, ethnic minority respondents and more educated participants (online supplemental table 4). Associations between drinking category and hypothetical behaviour changes are summarised in table 4. Notably, more hazardous than low-risk drinkers stated that they would change their behaviour if labelling was introduced (53.5% vs 44.4%), with adjusted odds of making changes of 1.43 (95% CI 1.199 to 1.699).

**Table 4:** Hypothetical responses to alcohol calorie labelling alcohol drinkers only

Response to labelling	Alcohol group	N (%)	Adjusted OR (95% CI)	P value
Make changes	Low-risk	1214 (44.4%)	1	<0.0001
	Hazardous	424 (53.5%)	1.43 (1.199 to 1.699)	
Drink fewer alcoholic drinks	Low-risk	350 (12.8%)	1	0.020
	Hazardous	133 (16.8%)	1.33 (1.045 to 1.685)	
Drink alcohol less often	Low-risk	374 (13.7%)	1	0.015
	Hazardous	140 (17.7%)	1.34 (1.058 to 1.689)	
Choose lower calorie alcoholic drinks	Low-risk	585 (21.4%)	1	0.009
	Hazardous	216 (27.0%)	1.31 (1.069 to 1.595)	
Choose smaller servings of alcoholic drinks	Low-risk	378 (13.8%)	1	0.90
	Hazardous	106 (13.4%)	0.98 (0.763 to 1.266)	
Eat smaller meals or fewer snacks	Low-risk	211 (7.7%)	1	0.75
	Hazardous	68 (8.6%)	1.10 (0.807 to 1.508)	
Do more exercise	Low-risk	344 (12.6%)	1	0.042
	Hazardous	140 (17.7%)	1.27 (1.009 to 1.606)	

Note: odds ratiosORs adjusted for age, gender, ethnicity, occupational class, and education.

Of the six types of possible behaviour change assessed, the most common was choosing lower calorie drinks, endorsed by 22.4% of alcohol drinkers, followed by drinking alcohol less often (14.5%), consuming fewer drinks (13.7%), doing more exercise (13.7%) and choosing smaller servings (13.3%). The least common response was eating smaller meals or fewer snacks (8.0%). The correlates of these responses are also summarised in online supplemental table 4. Positive responses were more common among women than men, except in the case of exercise where more men said they would increase exercise levels. Younger participants were more likely to endorse all items except for eating smaller meals, a pattern that was also reported in minority ethnic and more educated respondents. Associations with socioeconomic position were less consistent, but classes A/B and C1 were more likely to endorse changes in several behaviours than were classes C2 and D/E.

The differences between low-risk and hazardous drinkers in their hypothetical behavioural responses are summarised in table 4. Hazardous drinkers were more likely than low-risk drinkers to state that they would drink fewer alcoholic drinks (OR 1.33, 95% CI 1.045 to 1.685), that they would drink less often (OR 1.34, 95% CI 1.058 to 1.689), that they would choose lower calorie drinks (OR 1.31, 95% CI 1.069 to 1.595) and that they would do more exercise (OR 1.27, 95% CI 1.009 to 1.606) after adjustment for covariates. There were no differences between alcohol groups in other behaviour changes.

As might be anticipated, respondents who indicated that they would not change their drinking behaviour if alcohol calorie labelling was introduced were more negative in their attitudes to labelling. Compared with the remainder of alcohol drinkers, people who stated that they would not make changes had markedly reduced likelihoods of agreeing that labelling would be useful, should be introduced in shops and supermarkets, or should be introduced in pubs, bars and restaurants (adjusted ORs 0.26–0.35).

### Sensitivity analyses

The sensitivity analyses used the stricter definition of accuracy of estimating the calorie content of drinks. The proportion of participants who gave exact correct responses was substantially lower than with the broader definition, with only 8.0% being accurate for beer, 14.0% for white wine, 12.5% for cider and 18.6% for spirits (online supplemental table 5). Both underestimation and overestimation increased but overestimation remained much more common. The associations with alcohol drinking levels were generally reduced compared with the main analyses (online supplemental table 6). Nevertheless, hazardous drinkers remained more accurate than non-drinkers in estimating the calorie content of white wine and spirits, independently of covariates.

## Discussion

This study of a national sample of adults in England found that the majority agreed that alcohol calorie labelling would be useful, but both low-risk and hazardous drinkers were less positive than non-drinkers about the introduction of labelling in shops, supermarkets and hospitality venues. People who drank at hazardous levels were more accurate in their estimates of the calorie content of alcoholic beverages than were non-drinkers, with low-risk drinkers being intermediate. Just over half of drinkers reported that they would not change their drinking patterns in the event of alcohol calorie labelling, but this was more common among low-risk drinkers. If labelling was introduced, hazardous drinkers were more likely than low-risk drinkers to indicate that they would consume fewer drinks, drink less often, choose lower-calorie beverages and do more exercise.

There have been a number of studies of attitudes, knowledge and behaviour in relation to alcohol calorie labelling, but few of these have focused on differences in response related to typical alcohol consumption.^1521^,^23^ Contrasting low-risk and hazardous drinkers is relevant for several reasons. Hazardous drinkers consume more, so alcohol makes a greater contribution to total energy intake than it does for low-risk drinkers. If alcohol calorie labelling has any effect on calorie intake and risk of excessive body weight, it will be most evident in this group. Additionally, hazardous drinkers are at higher risk for alcohol-related harm.

The proportion of alcohol drinkers compared with non-drinkers was 75.3%, of whom 29.1% of men and 15.9% of women were classified as drinking in the hazardous to harmful range. The 2021 Health Survey for England reported comparable levels of consumption, with 28% of men and 15% of women drinking above recommended levels.^2^ Our finding of higher prevalence of hazardous drinking among more educated individuals and those in higher socioeconomic positions is also consistent with the Health Survey.

Attitudes to mandatory calorie labelling were generally positive, but were less positive among men, white British and older people, and those in higher education or occupational groups, particularly in relation to labelling in hospitality venues. Previous studies have been inconsistent in relating attitudes with sociodemographic characteristics.^7 11 12^ More negative opinions were associated with a lower likelihood of making changes in consumption were labelling to be introduced, suggesting that efforts to modify attitudes may be fruitful.

Previous studies estimating knowledge of calorie content have frequently involved asking people how many calories a glass of wine or pint of beer contains without providing any reference figures, and have tended to report underestimation of calories.^11 21^ Given public awareness about calories in general is limited, this low level of accuracy is unsurprising. We therefore provided ranges of calories rather than asking for specific values. Using this method, overestimation rather than underestimation was much more common (online supplemental table 2), as has previously been reported.^9 23^ Non-drinkers are more likely to say that they do not know calorie content (41.4%–42.3% for different beverages).

An important concern about awareness is whether drinkers, particularly hazardous drinkers, underestimate the calories in alcohol. If this were the case, then campaigns to inform the public about alcohol calories might raise awareness of the role of alcohol in total calorie content.^15^ However, we observed the opposite: people who consumed alcohol were more likely to be correct in their estimates than non-drinkers. This suggests that ignorance of calorie content is not a specific characteristic of hazardous drinkers.

We assessed hypothetical behaviour changes that might occur in response to mandatory alcohol calorie labelling. Just over half of drinkers stated that alcohol calorie labelling would make no difference to their behaviour. However, hazardous drinkers were more likely than low-risk drinkers to indicate that they would change their behaviour (53.5% vs 44.4%). Since low-risk drinkers consume relatively little alcohol, they may perceive little benefit to reducing their consumption. Hazardous drinkers endorsed a range of behaviour changes including consuming fewer drinks, choosing lower-calorie drinks, drinking less often and doing more exercise. Many of these behaviours have been noted in previous survey studies,^16^ but have not been related to levels of alcohol consumption. Although the proportion of alcohol consumers endorsing each of these responses was relatively small, this suggests that labelling may effectively target higher-risk drinkers who are obtaining a greater proportion of calories from alcohol. However, it is of concern that older and less educated alcohol consumers appear to be more resistant to making changes in response to alcohol calorie labelling.

### Study limitations

This was a self-report study, so responses may not correspond with actual behaviour. The amount of alcohol consumed may be incorrect because of inaccurate recall or deliberate misreporting, particularly among heavier drinkers.^24^ Although a large sample was recruited, the number of individuals who reported harmful levels of drinking was small. We did not have information about whether participants had experience with calorie counting, and this may account for some variations in the responses to the questions concerning calorie content. Season of the year was not taken into account, and results might have been different if data had been collected in the summer months.

A further limitation is that data on non-response rates are not available, so we do not know how many households were approached to derive the sample. Response rates are difficult to compute when households within randomly selected output areas are approached in order to complete quotas. However, comparisons in the context of smoking (another component of this survey) indicate that key variables such as sociodemographic and smoking characteristics are nationally representative.^25 26^

### Implications for policy

These results have several implications for policy. First, enhancement of knowledge about the calorie content of alcohol beverages through educating the public may be a lower priority than targeting attitudes. Our results indicate that alcohol drinkers are no less knowledgeable about calorie content than non-drinkers, and that hazardous drinkers are more aware of calories than low-risk or non-drinkers about calorie content. Overestimation of calorie content was common, so increasing accuracy might even encourage some drinkers to consume more.

Second, although attitudes to alcohol calorie labelling were generally positive, there was resistance to the practical implementation of such information. Educational gradients in attitudes suggest that ensuring information campaigns are accessible to less educated groups might pay dividends, and avoid widening of socioeconomic gradients in health risk.^27^ Third, the results suggest that increasing the availability of lower-calorie alcoholic beverages is desirable. The selection of lower alcohol alternatives was the most common action reported in response to mandatory labelling, and was particularly likely to be endorsed by hazardous drinkers. By contrast, the provision of lower-calorie snacks in hospitality venues is unlikely to be relevant according to our findings; few people said that they would select these options, and the proportion did not differ by drinking level. Finally, only around half of alcohol drinkers indicated that they would make changes if calorie labelling was introduced. It is therefore unlikely that on its own, alcohol calorie labelling will contribute substantially to the management of obesity or the UK Government aim to reduce alcohol-related harm. However, it might have a positive role as part of broader public health strategies, including reducing the availability of large serving sizes, taxation and price regulation.^7 16 28 29^

### Conclusions

A sizeable proportion of hazardous drinkers indicated that they would change their consumption pattern if mandatory calorie labelling was introduced, including consuming fewer drinks, drinking less often and choosing lower alcohol beverages. Underestimation of the caloric content of alcoholic beverages was uncommon, but drinkers were less positive than non-drinkers about the introduction of labelling in shops, supermarkets and hospitality venues. Policy efforts might usefully be directed towards promoting more positive attitudes to calorie labelling, and increasing the availability of lower-calorie beverages, to support reduced consumption of calories from alcoholic beverages at a population level.

## supplementary material

10.1136/bmjopen-2024-087491 online supplemental file 1

## Data Availability

Data are available upon reasonable request.
