# Supplementary material for: Potential impact of alcohol calorie labelling on the attitudes and drinking behaviour of hazardous and low-risk drinkers in England: a national survey
Source: BMJ Open. 2024 Aug 31;14(8):e087491. doi: 10.1136/bmjopen-2024-087491 (PMC11429355; doi:10.1136/bmjopen-2024-087491)
Supplement: online supplemental file 1 [file bmjopen-14-8-s001.pdf]

Potential Impact of Alcohol Calorie Labelling on the Attitudes and Drinking Behaviour of Hazardous and Low-Risk Drinkers in England: A National Survey

Supplementary Materials

Alcohol Calorie Labelling Study Questions

Question 1: Knowledge of calorie content in alcoholic drinks

**How many calories do you think the following alcoholic drinks contain? If you don't know, please provide your best estimate**	**50-99 kcals**	**100-149 kcals**	**150-199 kcals**	**200-249 kcals**	**250-300 kcals**
A medium glass of white wine (175ml 13% strength)					
A pint of beer (568ml 4% strength)					
A pint of cider (568ml, 4.5% strength)					
A single measure of gin or vodka (25ml)					

Question 2: Attitudes to calorie labelling on alcoholic drinks

**Please say to what extent you agree or disagree with the following:**	**Strongly disagree**	**Disagree**	**Neither agree nor disagree**	**Agree**	**Strongly agree**
It should be a legal requirement that calorie information is provided on alcoholic drinks purchased from shops.					
It should be a legal requirement that calorie information is provided for alcoholic drinks purchased in pubs, bars and restaurants					
I would find calorie labelling on alcoholic drinks useful					

Question 3: Perceived behavioural effects of calorie labelling

If calorie information was provided on alcoholic drinks, which one of the following would be most likely you do?

1. I wouldn't change my drinking.
2. I would drink fewer alcoholic drinks.
3. I would drink less often.
4. I would choose lower calorie alcoholic drinks.
5. I would choose smaller serving sizes of alcoholic drinks.
6. I would eat less (for example smaller meals or fewer snacks).
7. I would do more exercise.
8. None of these.
9. I don't know.

Supplement Table 1 Factors associated with attitudes to the introduction of alcohol calorie labelling

	Alcohol calorie labelling would be useful		Alcohol calorie labelling should be provided in shops and supermarkets		Alcohol calorie labelling should be provided in pubs, bars, and restaurants	
	N (%) agreement	p	N (%) agreement	p	N (%) agreement	p
Men	1262 (55.6%)	0.003	1426 (62.3%)	0.14	1181 (51.7%)	0.99
Women	1453 (60.0%)		1674 (64.4%)		1255 (51.7%)	
Age (yr)[1]	*r* = 0.043	0.004	*r* = -0.053	<0.001	*r* = -0.019	0.19
Ethnicity						
White British	2112 (56.4%)	<0.001	2321 (61.5%)	<0.001	1851 (49.3%)	<0.001
Other categories	603 (63.8%)		679 (70.7%)		585 (61.2%)	
Education qualifications						
None	436 (54.7%)	<0.0001	487 (60.3%)	<0.0001	427 (53.4%)	0.81
GCSE/O level	511 (54.5%)		574 (60.8%)		476 (50.5%)	
A level/equivalent	698 (57.1%)		758 (61.2%)		609 (49.5%)	
Degree	1070 61.8%)		1181 (67.8%)		924 (53.1%)	
Occupational class						
A/B	646 (60.0%)	0.013	720 (66.5%)	0.017	436 (52.4%)	0.82
C1	1025 (59.1%)		1115 (64.1%)		363 (50.3%)	
C2	401 (55.4%)		433 (59.9%)		899 (51.8%)	
D/E	450 (55.4%)		522 (62.4%)		436 (52.4%)	

[1] Point-biserial correlation

Supplement Table 2 Knowledge of calorie content of alcoholic beverages

	Knowledge category	Complete sample	Non-drinkers	Low-risk drinkers	Hazardous drinkers
**Beer (pint, 4% ABV)**	Underestimation	386 (8.2%)	121 (10.5%)	218 (8.0%)	47 (5.9%)
	Correct	1315 (28.1%)	240 (20.8%)	794 (29.0%)	281 (35.5%)
	Overestimation	1811 (38.7%)	316 (27.4%)	1140 (41.7%)	355 (44.8%)
	Don't know/refuse	1171 (25.0%)	478 (41.4%)	584 (21.3%)	109 (13.8%)
**Wine (175ml, 13% ABV)**	Underestimation	704 (15.0%)	177 (15.3%)	424 (15.5%)	103 (13.0%)
	Correct	1934 (41.3%)	318 (27.5%)	1190 (43.5%)	426 (53.8%)
	Overestimation	914 (19.5%)	178 (15.4%)	592 (21.6%)	144 (18.2%)
	Don't know/refuse	1131 (24.2%)	482 (41.7%)	530 (19.4%)	119 (15.0%)
**Cider (pint, 4.5% ABV)**	Underestimation	690 (14.7%)	185 (16.0%)	405 (14.8%)	100 (12.6%)
	Correct	1571 (33.5%)	249 (21.6%)	981 (35.9%)	341 (43.1%)
	Overestimation	1181 (25.2%)	232 (20.1%)	725 (26.5%)	224 (28.3%)
	Don't know/refuse	1241 (26.5%)	489 (42.3%)	625 (22.8%)	127 (16.0%)
**Sprits (25ml, 40% ABV)**	Underestimation	-	-	-	-
	Correct	2411 (51.5%)	438 (37.9%)	1411 (52.7%)	532 (67.2%)
	Overestimation	1043 (22.3%)	224 (19.4%)	679 (24.8%)	140 (17.7%)
	Don't know/refuse	1229 (26.2%)	493 (42.7%)	616 (22.5%)	120 (15.2%)

Supplement Table 3 Factors associated with correct estimates of alcoholic beverages

	**Beer (pint, 4% ABV)**		**White wine (175ml, 13% ABV)**		**Cider (pint, 4.5% ABV)**		**Spirits (25ml, 40% ABV)**	
	N (%)	P	N (%)	p	N (%)	p	N (%)	p
Men	687 (39.0%)	0.069	946 (55.1%)	0.55	769 (45.2%)	0.66	1177 (70.1%)	0.77
Women	678 (36.1%)		1061 (54.1%)		1007 (46.0%)		1321 (69.6%)	
Age (yr)[1]	*r* = 0.030	0.076	*r* = -0.028	0.096	*r* = -0.041	0.015	*r* = -0.047	0.006
Ethnicity								
White British	1116 (38.1%)	0.12	1647 (55.5%)	0.019	1359 (47.2%)	<0.0001	2025 (70.2%)	0.29
Other categories	249 (35.0%)		360 (50.6%)		268 (39.1%)		473 (68.2%)	
Education qualifications								
None	164 (37.2%)	0.34	193 (41.7%)	<0.0001	142 (33.3%)	<0.0001	299 (69.5%)	0.54
GCSE/O level	276 (39.9%)		371 (52.8%)		315 (46.8%)		482 (70.1%)	
A level/equivalent	382 (38.3%)		545 (54.8%)		425 (43.3%)		662 (68.1%)	
Degree	543 (36.0%)		898 (59.2%)		745 (50.2%)		1055 (70.9%)	
Occupational class								
A/B	335 (36.5%)	0.85	546 (58.8%)	<0.0001	442 (49.4%)	<0.0001	652 (72.0%)	0.15
C1	526 (37.5%)		810 (56.6%)		646 (46.7%)		956 (68.8%)	
C2	328 (40.1%)		293 (54.3%)		239(44.2%)		371 (69.6%)	
D/E	187 (34.6%)		235 (43.0%)		191 (36.3%)		354(68.1%)	

[1] Point-biserial correlation

Note: Analyses of respondents who provided an estimate

Supplement Table 4 Factors associated with hypothetical responses to alcohol calorie labelling

Alcohol drinkers only

	**I would change my drinking**		**Drink fewer alcoholic drinks**		**Drink alcohol less often**		**Choose lower calorie alcoholic drinks**	
	N (%)	P	N (%)	p	N (%)	p	N (%)	p
Men	783 (42.3%)	<0.0001	222 (13.0%)	0.002	236 (12.7%)	0.004	329 (17.8%)	<0.001
Women	958 (51.4%)		2884 (15.4%)		301 (16.1%)		502 (26.9%)	
Age (yr)[1]	*r* = -0.115	<0.00001	*r* = -0.090	<0.001	*r* = -0.064	<0.001	*r* = -0.156	<0.001
Ethnicity								
White British	1376 (44.3%)	<0.0001	402 (13.0%)	0.004	409 (13.2%)	<0.0001	604 (21.4%)	0.002
Other categories	365 (59.7%)		107 (17.5%)		128 (20.9%)		167 (27.3%)	
Education qualifications								
None	230 (40.7%)	<0.001	51 (9.0%)	<0.0001	67 (11.9%)	<0.001	84 (14.9%)	<0.0001
GCSE/O level	332 (45.3%)		91 (12.4%)		96 (13.1%)		155 (21.1%)	
A level/equivalent	421 (43.1%)		117 (12.0%)		127 (13.0%)		186 (19.0%)	
Degree	758 (52.6%)		251 (17.4%)		247 (17.1%)		406 (28.2%)	
Occupational class								
A/B	437 (48.6%)	0.056	150 (16.7%)	<0.001	133 (14.8%)	0.49	258 (28.7%)	<0.0001
C1	684 (47.1%)		196 (13.5%)		210 (14.5%)		318 (21.9%)	
C2	259 (44.7%)		57 (9.8%)		76 (13.1%)		112 (19.3%)	
D/E	233 (44.0%)		59 (11.2%)		74 (14.0%)		92 (17.4%)	

[1] Point-biserial correlation

Supplement Table 4 continued **Factors associated with hypothetical responses to alcohol calorie labelling**

Alcohol drinkers only

	**Choose smaller servings of alcohol**		**Eat smaller meals or fewer snacks**		**Do more exercise**	
	N (%)	P	N (%)	p	N (%)	p
Men	174 (9.4%)	<.0001	133 (7.2%)	0.090	278 (15.0%)	0.022
Women	322 (17.3%)		163 (8.7%)		231 (12.4%)	
Age (yr)[1]	r = -0.054	<0.0001	r = -0.016	0.32	r = -0.118	<0.0001
Ethnicity						
White British	388 (12.5%)	<0.001	237 (7.6%)	0.102	388 (12.5%)	<0.0001
Other categories	108 (17.7%)		59 (9.7%)		121 (19.8%)	
Education qualifications						
None	55 (9.7%)	<0.001	41 (7.3%)	0.15	55 (9.7%)	<0.001
GCSE/O level	83 (11.3%)		51 (7.0%)		96 (13.1%)	
A level/equivalent	124 (12.7%)		79 (8.1%)		135 (13.8%)	
Degree	234 (16.2%)		125 (8.7%)		223 (15.5%)	
Occupational class						
A/B	133 (14.8%)	0.002	69 (7.7%)	0.71	117 (13.0%)	0.79
C1	210 (14.5%)		116 (8.0%)		216 (14.9%)	
C2	701 (12.1%)		50 (8.6%)		80 (13.8%)	
D/E	50 (9.5%)		42 (7.9%)		67 (12.7%)	

[1] Point-biserial correlation

Supplement Table 5 Knowledge of calorie content of alcoholic beverages (strict definition)

	Knowledge category	Complete sample	Non-drinkers	Low-risk drinkers	Hazardous drinkers
**Beer (pint, 4% ABV)**	Underestimation	704 (15.0%)	189 (16.4%)	410 (15.0%)	105 (13.3%)
	Correct	374 (8.0%)	70 (6.1%)	216 (7.9%)	88 (11.1%)
	Overestimation	2434 (52.0%)	418 (36.2%)	1526 (55.8%)	490 (61.9%)
	Don’t know/refuse	1171 (25.0%)	478 (41.1%)	584 (21.3%)	109 (13.8%)
**Wine (175ml, 13% ABV)**	Underestimation	1330 (28.4%)	313 (27.1%)	788 (28.8%)	229 (28.9%)
	Correct	656 (14.0%)	92 (8.0%)	424 (15.5%)	140 (17.7%)
	Overestimation	1566 (33.4%)	268 (23.2%)	994 (36.3%)	304 (38.4%)
	Don’t know/refuse	1131 (24.2%)	482 (41.7%)	515 (19.4%)	119 (15.0%)
**Cider (pint, 4.5% ABV)**	Underestimation	1031 (22.0%)	243 (21.0%)	616 (22.5%)	172 (21.7%)
	Correct	585 (12.5%)	102 (8.8%)	356 (13.0%)	127 (16.0%)
	Overestimation	1826 (39.0%)	321 (27.8%)	1139 (41.6%)	366 (46.2%)
	Don’t know/refuse	1241 (26.5%)	489 (42.3%)	625 (22.8%)	127 (16.0%)
**Sprits (25ml, 40% ABV)**	Underestimation	780 (16.7%)	160 (13.9%)	441 (16.1%)	179 (22.6%)
	Correct	872 (18.6%)	136 (11.8%)	518 (18.9%)	218 (27.5%)
	Overestimation	1802 (38.5%)	366 (31.7%)	1161 (42.4%)	275 (34.7%)
	Don’t know/refuse	1229 (26.2%)	493 (42.7%)	616 (22.5%)	120 (15.2%)

Supplement Table 6 Knowledge of calorie content of alcoholic beverages (exact definition)

Beverage	Alcohol group	Correct estimate N (%)	Adjusted odds ratio (95% CI)	p
Beer (pint, 4% ABV)	Non-drinkers	70 (10.3%)	1	
	Low-risk	216 (10.0%)	0.93 (0.687-1.269)	0.66
	Hazardous	88 (12.9%)	1.12 (0.781-1.616)	0.53
White wine (175ml, 13% ABV)	Non-drinkers	92 (13.7%)	1	
	Low-risk	424 (19.2%)	1.38 (1.056-1.802)	0.018
	Hazardous	140 (20.8%)	1.40 (1.399-1.919)	0.039
Cider (pint, 4.5% ABV)	Non-drinkers	102 (15.3%)	1	
	Low-risk	356 (16.9%)	1.08 (0.824-1.383)	0.62
	Hazardous	127 (19.1%)	1.21 (0.994-1.645)	0.23
Spirits (25ml, 40% ABV)	Non-drinkers	136 (20.5%)	1	
	Low-risk	518 (24.4%)	1.21 (0.959-1.517)	0.11
	Hazardous	218 (32.4%)	1.83 (1.399-2.392)	<0.0001

Note: odds ratios adjusted for age, gender, ethnicity, occupational class, and education

Analysis of respondents who provided an estimate.
