# Supplementary material for: Potential impact of alcohol calorie labelling on the attitudes and drinking behaviour of hazardous and low-risk drinkers in England: a national survey
Source: BMJ Open. 2024 Aug 31;14(8):e087491. doi: 10.1136/bmjopen-2024-087491 (PMC11429355; doi:10.1136/bmjopen-2024-087491)
Supplement: Supplementary file 2 [file bmjopen-2024-087491.reviewer_comments.pdf]

PEER REVIEW HISTORY

BMJ Open publishes all reviews undertaken for accepted manuscripts. Reviewers are asked to complete a checklist review form and are provided with free text boxes to elaborate on their assessment. These free text comments are reproduced below.

ARTICLE DETAILS

**TITLE (PROVISIONAL)**	Potential Impact of Alcohol Calorie Labelling on the Attitudes and Drinking Behaviour of Hazardous and Low-Risk Drinkers in England: A National Survey
**AUTHORS**	Steptoe, Andrew; Sheen, Florence; Conway, Rana; Llewellyn, Clare; Brown, Jamie

VERSION 1 - REVIEW

**REVIEWER NAME**	Foster, John
**REVIEWER AFFILIATION**	University of Greenwich, London, UK, Psychology, Social Work and Counselling
**REVIEWER CONFLICT OF INTEREST**	None
**DATE REVIEW RETURNED**	11-May-2024

**GENERAL COMMENTS**	I greatly enjoyed reading this paper and I feel it is well written and largely methodologically robust but I have some minor suggestions for the authors to consider.<br><br>Strengths and Limitations:<br><br>I suggest these are separated in the strengths and limitations page.<br><br>Quota Sampling: Are there any details of the non-respondents contacted to get to the 4,901 respondents. If not please state this and include it as a limitation (see comment below).<br><br>The discussion does not include the strengths and limitations and this should be included and follow the 5 bullet points already presented. I would also add a comment about quota sampling. As I understand it you cannot estimate the non-responders and thus how representative the sample is.

**REVIEWER NAME**	Joseph, Paule Valery
**REVIEWER AFFILIATION**	National Institute on Alcohol Abuse and Alcoholism
**REVIEWER CONFLICT OF INTEREST**	None
**DATE REVIEW RETURNED**	20-May-2024

**GENERAL COMMENTS**	Using a cross-sectional national telephone survey involving 4,683 adult participants in England from November 2022 to January 2023, the authors sought to examine the potential effects of mandatory alcohol calorie labeling on various groups: non-drinkers, low-risk drinkers, and hazardous drinkers. The authors also evaluated the individuals attitudes, knowledge of alcohol's calorie content, and

	hypothetical changes in drinking behavior in response to calorie labeling. Overall, it is an interesting concept; however, there are a few major issues that must be addressed before publication. Proper attribution must be provided throughout especially the discussion.<br><br>1. It would be helpful if the authors explicitly stated their hypothesis in page 7<br><br>2. It is unclear if participants had prior knowledge about calorie counting - what was their literacy on the matter?<br><br>3. A large part of the discussion is a repetition of the results rather than discussing the implications of these results page 13 line 49-59, pag 14 line 3-59, page 15 loine 3-15. It would be helpful if the authors discuss the implications of these findings and how they compare or not to work prior<br><br>4. Many large sections of the discussion without attribution or proper citation '<br><br>5. It would be helpful if the authors add implications for clinical practice as well.<br><br>6. In the conclusions, line 54-59, overestimate the findings - it would be sensible to state that based on this survey hypothetically....<br><br>Tables need explanations for abreviations

**REVIEWER NAME**	Harrison, Nathan
**REVIEWER AFFILIATION**	Flinders University
**REVIEWER CONFLICT OF INTEREST**	None
**DATE REVIEW RETURNED**	25-May-2024

**GENERAL COMMENTS**	Thank you for the opportunity to review this interesting manuscript. Labelling interventions have received attention in several countries, and understanding consumers' potential reactions is important to inform policy implementation. This is research based on a large national sample, presented in a very clear and engaging manuscript.<br>The manuscript focuses on hypothetical behaviour changes, as noted in the Abstract – so I would suggest making this clear in the title (i.e., 'Impact of Alcohol Calorie Labelling on… Drinking Behaviour' might imply that the actual performance or change of behaviour has been studied).<br>The Introduction covers existing literature succinctly and effectively. There are times when it would further strengthen the rationale to make clear what the target of labelling interventions is/is expected to be (e.g., with 'small benefits' on ms p4 line 45) – are these focused on reduced alcohol intake and/or reduced caloric intake? Similarly, there a few cases where extra precision would helpful (e.g., p12 l24 – light levels of drinking among the group presumably, rather than drinkers with lower weight).<br>In the Methods, I would suggest specifying the mid-point of the 1-5 anchor (i.e., the value of 3) – the appropriateness of collapsing this into the 'disagreement' category is not currently clear.<br>The manuscript also notes (quite appropriately) that because understanding of calorie content appears to be high in this sample, labelling interventions might not work via information provision but

	rather through reinforcing awareness, influencing attitudes, or similar. My main concern about the implications of providing lower-calorie alcoholic drinks is that this would not reduce alcohol-related harms, in itself – although I appreciate that this could contribute to reduced energy intake (and have subsequent benefits). In contrast, could reducing the availability of large serving sizes (which is mentioned later in the Discussion, but not framed as a final Conclusion) seemingly do both? Given that the effects of labelling are likely to be relatively modest, the authors could also comment briefly on the broader policies with potential to have more substantial population-level impacts, including any policies that may reinforce or otherwise complement labelling (e.g., labelling based on potential health harms).<br>I also offer the following minor suggestions for consideration:<br>- There appear to be some associations that get lost in the current reporting. At p10 l43 / supplementary table 4 - doing more exercise seems to be significantly higher among men (but is described overall, that ‘Positive responses were more common among women’); similarly, the association with age is not significant for eating smaller meals or fewer snacks.<br>- Apologies if I’ve missed something, but I don’t understand why the seemingly significant differences in Table 4 on ‘make changes’ and ‘do more exercise’ are not explained with the other significant differences.<br>- Regarding Table 2’s description in-text, it isn’t clear if unadjusted analyses are being described at p9 l8 – and if so, if these are presented in a supplementary table or elsewhere. There are several other instances of this (e.g., p10 l8, p11 l8-20 – could the authors please refer to where this data is available?).<br>- I feel the tables would more effectively stand alone with N’s for total sample and total subgroups (non-drinkers, alcohol drinkers…), that (presumably) means for age and AUDIT scores are presented, etc.<br>- p12 l31 – ‘compared with non-drinkers’ suggests the figure for non-drinking % is mentioned here, when it is does not appear to be.<br>- The manuscript makes study strengths and limitations clear. It’s also worth considering if respondents could change consumption in other ways not explicitly captured in the chosen items. For example, respondents may also avoid drinking at particular times of the year (with potential seasonal effects relevant given data collection in November-January).

VERSION 1 – AUTHOR RESPONSE

Reviewer: 1
Dr. John Foster, University of Greenwich, London, UK
Comments to the Author:
I greatly enjoyed reading this paper and I feel it is well written and largely methodologically robust but I have some minor suggestions for the authors to consider.

Strengths and Limitations:

I suggest these are separated in the strengths and limitations page.

We have added a section to the Discussion (pages 14-15 XX) detailing methodological limitations

Quota Sampling: Are there any details of the non-respondents contacted to get to the 4,901 respondents. If not please state this and include it as a limitation (see comment below).

Unfortunately, we do not have this information. The survey company responsible for data collection argue that non-response rates are not recorded because these are uninformative when homes within output areas are selected by interviewers in order to reach quotas rather than at random (i.e., they target people who are more difficult to reach). We agree that the hybrid of random (at output area-level) and quota (at individual-level) sampling is a limitation, but that comparisons in the context of smoking with other national random probability surveys and sales data indicate that key variables such as sociodemographic and smoking characteristics are nationally representative (Fidler JA, Shahab L, West O, et al. 'The smoking toolkit study': a national study of smoking and smoking cessation in England. BMC Public Health. 2011;11:479. doi:10.1186/1471-2458-11-479 ; Jackson SE, Beard E, Kujawski B, et al. Comparison of Trends in Self-reported Cigarette Consumption and Sales in England, 2011 to 2018. JAMA Netw Open. 2019;2(8):e1910161.). We have added to the limitations section (page 15) detailing this issue.

Reviewer: 2
Dr. Paule Valery Joseph, National Institute on Alcohol Abuse and Alcoholism
Comments to the Author:
Using a cross-sectional national telephone survey involving 4,683 adult participants in England from November 2022 to January 2023, the authors sought to examine the potential effects of mandatory alcohol calorie labeling on various groups: non-drinkers, low-risk drinkers, and hazardous drinkers. The authors also evaluated the individuals attitudes, knowledge of alcohol's calorie content, and hypothetical changes in drinking behavior in response to calorie labeling. Overall, it is an interesting concept; however, there are a few major issues that must be addressed before publication. Proper attribution must be provided throughout especially the discussion.

1. It would be helpful if the authors explicitly stated their hypothesis in page 7

We have added our hypothesis on page 7

2. It is unclear if participants had prior knowledge about calorie counting - what was their literacy on the matter?

No, we do not know of participants' experience of calorie counting, and have added this as a limitation on page 14.

3. A large part of the discussion is a repetition of the results rather than discussing the implications of these results page 13 line 49-59, pag 14 line 3-59, page 15 loine 3-15. It would be helpful if the authors discuss the implications of these findings and how they compare or not to work prior.

We have modified the section on page 13 to avoid duplication. But we are reluctant to shorten the sections on pages 14 and 15 (now 15-16) because they outline the implications for policy which are a central driver for this research.

4. Many large sections of the discussion without attribution or proper citation '

We have added additional citations to the Discussion section. We have referred readers to relevant reviews, because some topics (e.g. attitudes to calorie labelling) have been quite extensively studied by many researchers, and there is a limit to the number of references permitted by the journal.

5. It would be helpful if the authors add implications for clinical practice as well.

The context of this study was policy related to living with obesity rather than clinical alcohol problems, but we have noted both in the Discussion (page xx).

6. In the conclusions, line 54-59, overestimate the findings - it would be sensible to state that based on this survey hypothetically....

We appreciate this point, but would argue that the entire paper is framed in the context of hypothetical behaviours. This is made clear in the title and text, but we have now added ‘hypothetical’ to the Abstract to make this point completely clear.

Tables need explanations for abbreviations

We have added explanations of the socioeconomic and educational classifications to page XX, and other abbreviations to the Table legends.

Reviewer: 3
Mr. Nathan Harrison, Flinders University

Thank you for the opportunity to review this interesting manuscript. Labelling interventions have received attention in several countries, and understanding consumers’ potential reactions is important to inform policy implementation. This is research based on a large national sample, presented in a very clear and engaging manuscript.

The manuscript focuses on hypothetical behaviour changes, as noted in the Abstract – so I would suggest making this clear in the title (i.e., ‘Impact of Alcohol Calorie Labelling on… Drinking Behaviour’ might imply that the actual performance or change of behaviour has been studied).

We have added ‘hypothetical’ to make this clear.

The Introduction covers existing literature succinctly and effectively. There are times when it would further strengthen the rationale to make clear what the target of labelling interventions is/is expected to be (e.g., with ‘small benefits’ on ms p4 line 45) – are these focused on reduced alcohol intake and/or reduced caloric intake? Similarly, there a few cases where extra precision would helpful (e.g., p12 l24 – light levels of drinking among the group presumably, rather than drinkers with lower weight).

Thank you for noting this ambiguity in wording, which we have endeavoured to rectify.

In the Methods, I would suggest specifying the mid-point of the 1-5 anchor (i.e., the value of 3) – the appropriateness of collapsing this into the ‘disagreement’ category is not currently clear.

The manuscript also notes (quite appropriately) that because understanding of calorie content appears to be high in this sample, labelling interventions might not work via information provision but

rather through reinforcing awareness, influencing attitudes, or similar. My main concern about the implications of providing lower-calorie alcoholic drinks is that this would not reduce alcohol-related harms, in itself – although I appreciate that this could contribute to reduced energy intake (and have subsequent benefits). In contrast, could reducing the availability of large serving sizes (which is mentioned later in the Discussion, but not framed as a final Conclusion) seemingly do both? Given that the effects of labelling are likely to be relatively modest, the authors could also comment briefly on the broader policies with potential to have more substantial population-level impacts, including any policies that may reinforce or otherwise complement labelling (e.g., labelling based on potential health harms).

The mid-point of the attitude scales was labelled 'neither agree or disagree' which we decided to group with disagreement ratings, because our interest was in the prevalence of positive attitudes. We have now made this clearer on page 6. We agree with the reviewer about broader policies, and have modified the discussion of policy implications (page 15-16).

I also offer the following minor suggestions for consideration:

- There appear to be some associations that get lost in the current reporting. At p10 l43 / supplementary table 4 - doing more exercise seems to be significantly higher among men (but is described overall, that 'Positive responses were more common among women'); similarly, the association with age is not significant for eating smaller meals or fewer snacks.

- The reviewer is quite right that we did not mention these associations in the interests of space, and only made higher level comments on these responses. We have now extended the section om page 11 to detail these other associations.

- Apologies if I've missed something, but I don't understand why the seemingly significant differences in Table 4 on 'make changes' and 'do more exercise' are not explained with the other significant differences.

- The 'make changes' responses are discussed before all the others on page 10, because that is a global response. We neglected to include the 'do more exercise' response for hazardous drinkers, but that was an error. The alteration has now been made (page 11). We have also modified the Abstract to add this item.

- Regarding Table 2's description in-text, it isn't clear if unadjusted analyses are being described at p9 l8 – and if so, if these are presented in a supplementary table or elsewhere. There are several other instances of this (e.g., p10 l8, p11 l8-20 – could the authors please refer to where this data is available?).

All the % are unadjusted, while the ORs are adjusted. This is made clear in the legends to the tables, but to make this more clear we have added a phrase to the Method section (Page 8).

-

- I feel the tables would more effectively stand alone with N's for total sample and total subgroups (non-drinkers, alcohol drinkers…), that (presumably) means for age and AUDIT scores are presented, etc.

- We have not included Ns for all the analyses in the interests of clarity, but they can be computed from the Ns of the groups.

-

- p12 l31 – ‘compared with non-drinkers’ suggests the figure for non-drinking % is mentioned here, when it is does not appear to be.

- We have already noted the number of non-drinkers on page 8 and in the Abstract. This particular sentence is specifically concerned with the proportions of low-risk and hazardous drinkers.

-

- The manuscript makes study strengths and limitations clear. It’s also worth considering if respondents could change consumption in other ways not explicitly captured in the chosen items. For example, respondents may also avoid drinking at particular times of the year (with potential seasonal effects relevant given data collection in November-January).

- We agree with this point, and have added it to the Limitations section on page 15.

VERSION 2 – REVIEW

**REVIEWER NAME**	Harrison, Nathan
**REVIEWER AFFILIATION**	Flinders University
**REVIEWER CONFLICT OF INTEREST**	None
**DATE REVIEW RETURNED**	15-Jul-2024

**GENERAL COMMENTS**	The authors have addressed comments from the previous round of review with concise manuscript edits. Thank you and best wishes.

VERSION 2 – AUTHOR RESPONSE
